# A survey on the degree of eye discomfort caused by video terminal use among college students in different altitudes

**DOI:** 10.1186/s12889-023-16004-z

**Published:** 2023-06-22

**Authors:** Bingjie Liu, Daijiao Zhou, Zuyou Li, Yao Wang, Zhen Chen

**Affiliations:** 1 The Affiliated Hospital of Kunming University of Science and Technology, Kunming, 650500 P.R. China; 2 Department of Ophthalmology, The First People’s Hospital of Yunnan Province, Kunming, 650500 P.R. China; 3 Medical School, Kunming University of Science and Technology, Kunming, 650500 P.R. China

**Keywords:** Dry eye, college students, Altitude, Video display terminal (VDT), Tear film, Sleep disorders, Eye discomfort

## Abstract

**Objective:**

To analyze the risk factors associated with different levels of eye discomfort due to video terminal use among college students at different altitudes.

**Methods:**

This cross-sectional study was conducted to assess the prevalence and extent of eye discomfort by distributing an questionnaire to university students via the Internet. To analyze the causes and risk factors of eye discomfort among college students at different altitudes after using video terminals.

**Results:**

A total of 647 participants who met the criteria were included in this survey, of whom 292 (45.1%) were males and 355 (54.9%) were females. The results of the survey showed 194 (30.0%) participants without eye discomfort and 453 (70.0%) participants with eye discomfort. The results of the univariate comparison of the degree of eye discomfort in the study subjects with different characteristics showed that the differences in the degree of eye discomfort were statistically significant (P < 0.05) for the 7 groups of indicators: gender, region, wearing corneal contact lenses for more than 2 h per day, frequent use of eye drops, sleep time, total time of VDT use per day, and total time per VDT use, while the remaining indicators, including age, profession, and whether refractive surgery or other eye surgery was performed, whether frame glasses were worn for a long time, and duration of daily mask wear were not statistically significant. The results of multi-factor logistic analysis of the degree of eye discomfort in the study subjects with different characteristics showed that gender, region, frequent use of eye drops, sleep time, and total time of VDT use per day were the risk factors affecting the degree of eye discomfort.

**Conclusions:**

Female, high altitude, frequent use of eye drops, shorter daily sleep duration and longer daily VDT use were associated risk factors for the development of severe eye discomfort, where the severity of eye discomfort was significantly negatively correlated with increased sleep duration and significantly positively correlated with increased total time of VDT use.

## Introduction

The diseases that affect people’s eyes and physical and mental health due to the prolonged and frequent use of video terminals are collectively known as video display terminal syndrome (VDTs) and also known as computer vision syndrome (CVS) by the American Academy of Optometry [1–3]. This is a clinical syndrome that is characterized by dry eyes, visual fatigue, blurred vision, decreased visual acuity, itchy eyes, burning foreign body sensation, orbital pain, eye swelling and other ocular symptoms; dizziness, headache, nausea, sleep disturbance, memory loss, hair loss and other neurological symptoms; numbness, abnormal sensation, tremor, pressure pain, low back pain and other symptoms. numbness, abnormal sensation, tremor, pressure pain, low back pain and other shoulder, neck and wrist syndromes; loss of appetite, resistance, constipation, etc., and even different degrees of impact on the endocrine system.

Many studies have shown that eye-related symptoms with eye fatigue, irritation, burning sensation, dry eyes, redness, blurred vision, and other eye discomfort are the most common health problems among VDT users, and dry eyes seem to be the main influencing factor [1–12]. In recent years, due to the widespread use of video display terminal (VDT) such as cell phones, tablets, and computers, global warming, the popularity of air conditioning, and the aging of the population, the incidence of dry eye has increased significantly and has a tendency to become younger, with a prevalence of 30–50% worldwide and a prevalence of 21–30% in China. The number of dry eye patients is estimated to have reached 360 million, with a possible addition of 10% each year, and dry eye patients in China have accounted for more than 30% of ophthalmology outpatients, and dry eye has become an important problem affecting national eye health in China [9–16]. Dry eye and video terminal syndrome are closely related, and prolonged use of video terminals may lead to the gradual progression of functional dry eye with VDTs as the main manifestation to typical dry eye with physical signs [17, 18].

Video display terminal syndrome(VDTs)not only affect the quality of life of patients and have an impact on physical health, but also on mental health to varying degrees, and the global epidemic of the new crown epidemic has significantly exacerbated these discomfort symptoms and disease progression, leading to an increased economic burden on patients and society and seriously affecting the development of society [19–21].

As a growing public health problem, the factors affecting eye discomfort are multifaceted [22]. Our team’s preliminary investigation found that the prolonged use of video terminals (VDT) and the shortened sleep time were significantly correlated with the prevalence of eye discomfort symptom in high-altitude areas [23]. China is a vast country with a high topography in the west and a low topography in the east, and there are significant differences in altitude between different provinces [24]. It has been reported in the literature that environmental conditions such as low oxygen, low humidity, high wind, and strong ultraviolet light caused by high altitude can affect the ocular surface condition [25]. However, there are still no reports on the exact relationship between different altitudes and the degree of eye discomfort.

The global outbreak of the neo-crown epidemic in late 2019 has changed people’s lifestyles to varying degrees and has affected all aspects of our lives. In response to the national call to reduce the mobility of people to prevent the further spread of the rampant New Coronavirus, most university students have switched from offline face-to-face instruction to online teaching mode, and many of them were advised to strictly limit the frequency and scope of outdoor activities during the epidemic. Compared with the pre-epidemic period, this online based teaching mode with reduced offline exposure has undoubtedly greatly increased the time and opportunities to use video terminals during the learning process, and also leads to more attention to streaming video content or participation in entertainment activities such as video games, which leads to a significant increase in the likelihood of VDTs among school students as they spend more time looking at the screen [26–28].

China has a population of nearly 1.5 billion people, and according to the National Education Development Statistical Bulletin 2020 published by the Ministry of Education, there are more than 41 million university students in school, including a total of more than 1.1 million graduate students [29]. The group of enrolled college students has their own typical characteristics: most of them have passed the entrance physical examination and are relatively healthy without other underlying diseases; they have a higher education level and have completed 12 years of basic education, which enables them to complete the questionnaire effectively; their spare time and coursework require more time to use VDT compared with primary and secondary school students [30], so the subjects of this survey are chosen to be enrolled college students.

Quantitative assessment of patients’ subjective symptoms through questionnaires is considered to be one of the main methods for DED diagnosis [31–33]. The Ocular Surface Disease Index (OSDI) questionnaire is one of the most accurate specific questionnaires for ocular discomfort symptoms and is also an effective method commonly used for rapid diagnosis of DED [33–35]. Combined with the previous survey study, we adjusted the questions of the questionnaire based on the OSDI scale to fully understand the relevant factors affecting the degree of eye discomfort.

In this study, we selected college students from different altitudes in China as the subjects, and analyzed the risk factors related to the prevalence and degree of eye discomfort after using VDT in college students from different altitudes by filling out the questionnaire.

## Information and methods

### Survey subjects

A total of 647 college students from different altitudes in China were randomly selected to fill out the questionnaire, 292 were male students and 355 were female students, with an average age of 22.9 ± 4.9 years.

### Questionnaire content and assessment criteria

Before the start of the survey, participants were instructed to complete the questionnaire under a unified measurement standard right. The scores of the OSDI scale questions in the questionnaire were summed and then grouped according to the different total scores. The groups were divided into 4 groups according to the degree of eye discomfort symptom: no eye discomfort symptom group; mild eye discomfort symptom group; moderate eye discomfort symptom group; and severe eye discomfort symptom group. In this survey, based on the OSDI scale, additional questions were asked about the participants’ region, their university majors, whether they had refractive surgery or other eye surgery, whether they wore corneal contact lenses for more than 2 h per day, whether they wore frames for a long time, whether they used eye drops frequently, sleep time, total time of VDT use per day, and total time per VDT use, and the daily duration of wearing masks. This study was conducted to analyze the risk factors associated with the occurrence of eye discomfort and the degree of eye discomfort among college students at different altitudes.

The study was conducted by sharing the link to the online questionnaire through the Internet and inviting university students in different regions to participate in the survey voluntarily and anonymously. Before the release of the questionnaire, participants were informed of the requirements and details of the questionnaire in general on the linked platform. After clarifying that the survey was strictly confidential, participants chose whether to participate in the survey and gave their verbal informed consent. The study was approved by the Ethics Committee of the First People’s Hospital of Yunnan Province (KHLL2022-KY056) and was conducted in accordance with the principles of the Declaration of Helsinki. In accordance with the recommendations of the OSDI guidelines [34], all participants were informed of the requirement to fill in their comprehensive profile for the past week truthfully before completing the questionnaire, and all data were subsequently exported and analyzed via an Excel spreadsheet.

### Statistical methods

SAS 9.4 was used for data management and statistical analysis. Qualitative data were described using N(%), and the chi-square test and Fisher’s exact probability method were used for comparison between groups. For data obeying normal distribution,$\overline{\text{x}}\pm \text{s}$ was used for description and F test was used for comparison between groups; for quantitative data not obeying normal distribution, M (P25, P75) was used for description and rank sum test was used for comparison between groups. Ordinal logistic regression analysis was used to explore the factors influencing the degree of dry eye disease. The test level α = 0.05, and if there are not otherwise statement, the differences were considered statistically significant when *P* < 0.05.

## Results

### Description of the general characteristics of the study subject

A total of 647 subjects meeting the criteria were included in this study, with a mean age of 22.9 ± 4.9 years. Among them, 292 (45.1%) were male and 355 (54.9%) were female; 194 (30.0%) were without eye discomfort and 453 (70.0%) were with eye discomfort. The rest were detailed in Table 1.

**Table 1:** Description of the general characteristics of the study subjects

Variable	$N(\% )/\bar x \pm s$
**Gender**	
Male	292(45.1)
Female	355(54.9)
**Age**	22.9 ± 4.9
**Speciality**	
medical speciality	285(44.0)
Non-medical specialty	362(56.0)
**Region**	
Middle region	223(34.5)
East region	273(42.2)
West region	151(23.3)
**Perform refractive surgery or other eye surgery**	
Yes	45(7.0)
No	602(93.0)
**Wearing contact lenses for more than 2 h per day**	
Yes	54(8.3)
No	593(91.7)
**Wearing frames for a long time**	
Yes	290(44.8)
No	309(47.8)
Occasionally	48(7.4)
**Frequent use of eye drops**	
Yes	49(7.6)
No	598(92.4)
**Sleep time(h)**	7.4 ± 1.1
**Total time of VDT use per day(h)**	7.7 ± 3.6
**Total time per VDT use(h)**	2.9 ± 2.5
**The daily duration of wearing masks(h)**	5.5 ± 5.1
**Total score**	13.9 ± 13.5
**The degree of eye discomfort**	
**No eye discomfort**	194(30.0)
**Mild eye discomfort**	295(45.6)
**Moderate eye discomfort**	142(22.0)
**Severe eye discomfort**	16(2.4)

### Univariate analysis of the degree of eye discomfort in the study subjects with different characteristics

The results of the univariate comparison of the degree of eye discomfort in study subjects with different characteristics showed that the differences in the degree of eye discomfort were statistically significant for the 7 groups of indicators: gender, region, wearing corneal contact lenses for more than 2 h per day, frequent use of eye drops, sleep time, total time of VDT use per day, and total time per VDT use, and the remaining indicators including age, their university majors, whether or not refractive surgery or other eye surgery, whether or not to wear frame glasses for a long time, and the daily duration of wearing masks were not statistically significant and may not affect the degree of eye discomfort, as shown in Table 2.

**Table 2:** Comparison of the degree of eye discomfort in study subjects with different characteristics [n(%)]

Variable	No(*n* = 194)	Mild(*n* = 295)	Moderate(*n* = 142)	Severe(*n* = 16)	*χ* ^*2*^	*P*
**Gender**					22.98	< 0.001
Male	115(39.38)	118(40.41)	52(17.81)	7(2.40)		
Female	79(22.25)	177(49.86)	90(25.35)	9(2.54)		
**Speciality**					7.62	0.055
medical speciality	80(28.07)	126(44.21)	67(23.51)	12(4.21)		
Non-medical specialty	114(31.49)	169(46.69)	75(20.72)	4(1.10)		
**Region**					—	< 0.001
Middle region	84(37.67)	94(42.15)	36(16.14)	9(4.04)		
East region	81(29.67)	132(48.35)	57(20.88)	3(1.10)		
West region	29(19.20)	69(45.70)	49(32.45)	4(2.65)		
**Perform refractive surgery or other eye surgery**					—	0.205*
Yes	14(31.11)	16(35.56)	15(33.33)	0(0.00)		
No	180(29.90)	279(46.35)	127(21.10)	16(2.66)		
**Wearing contact lenses for more than 2 h per day**					—	0.050*
Yes	9(16.67)	27(50.00)	15(27.78)	3(5.56)		
No	185(31.20)	268(45.19)	127(21.42)	13(2.19)		
**Wearing frames for a long time**					—	0.294*
Yes	92(31.72)	132(45.52)	58(20.00)	8(2.76)		
No	94(30.42)	138(44.66)	69(22.33)	8(2.59)		
Occasionally	8(16.67)	25(52.08)	15(31.25)	0(0.00)		
**Frequent use of eye drops**					—	< 0.001*
Yes	3(6.12)	17(34.69)	27(55.10)	2(4.08)		
No	191(31.94)	278(46.49)	115(19.23)	14(2.34)		
**Sleep time(h)**	21.0(20.0,27.0)	21.0(19.0,26.0)	21.0(19.0,27.0)	25.0(19.5,29.0)	3.36	0.339#
**Total time of VDT use per day(h)**	8.0(7.0,8.0)	7.0(7.0,8.0)	7.0(7.0,8.0)	6.0(6.0,7.5)	17.56	< 0.001#
**Total time per VDT use(h)**	6.0(4.0,9.0)	8.0(5.0,10.0)	8.0(6.0,10.0)	10.0(8.0,11.5)	30.49	< 0.001#
**The daily duration of wearing masks(h)**	2.0(1.0,4.0)	2.0(1.0,4.0)	2.0(1.0,4.0)	3.0(2.0,8.0)	9.03	0.029#
**Frequent use of eye drops**	3.0(1.0,9.0)	4.0(1.0,8.0)	4.0(2.0,9.0)	8.0(2.5,12.0)	4.99	0.173#

Note: “*” used Fisher’s exact probability method; “#” used rank sum test

### Multi-factor logistic analysis of the degree of eye discomfort in study subjects with different characteristics

**Fig. 1:**
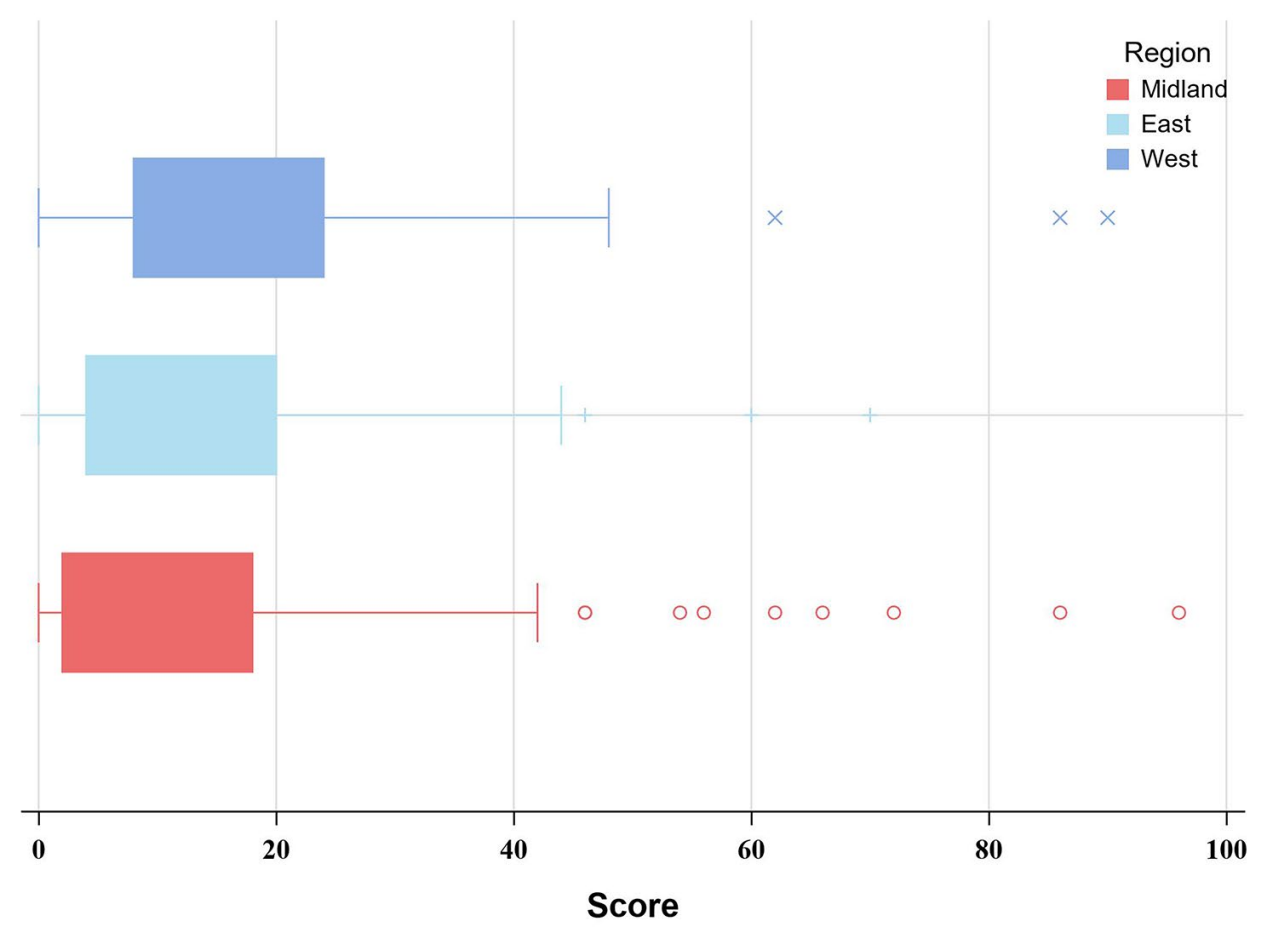
Total eye discomfort symptoms score with different regions

**Fig. 2:**
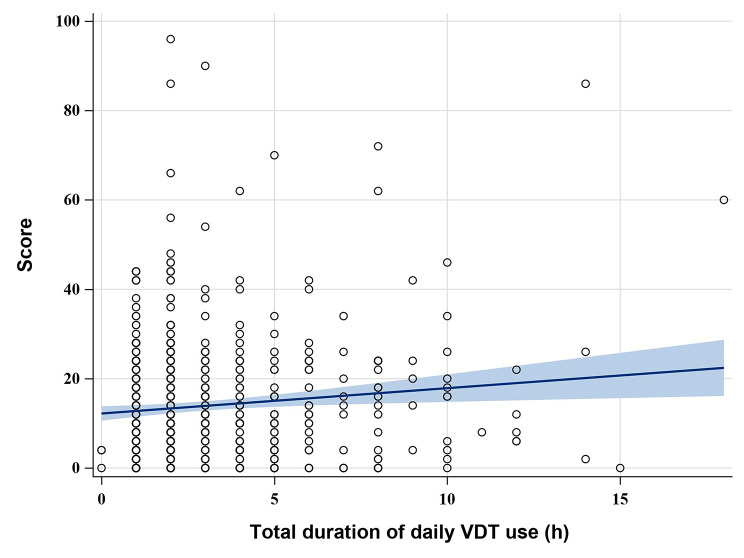
Total eye discomfort symptom score and daily VDT use time

**Fig. 3:**
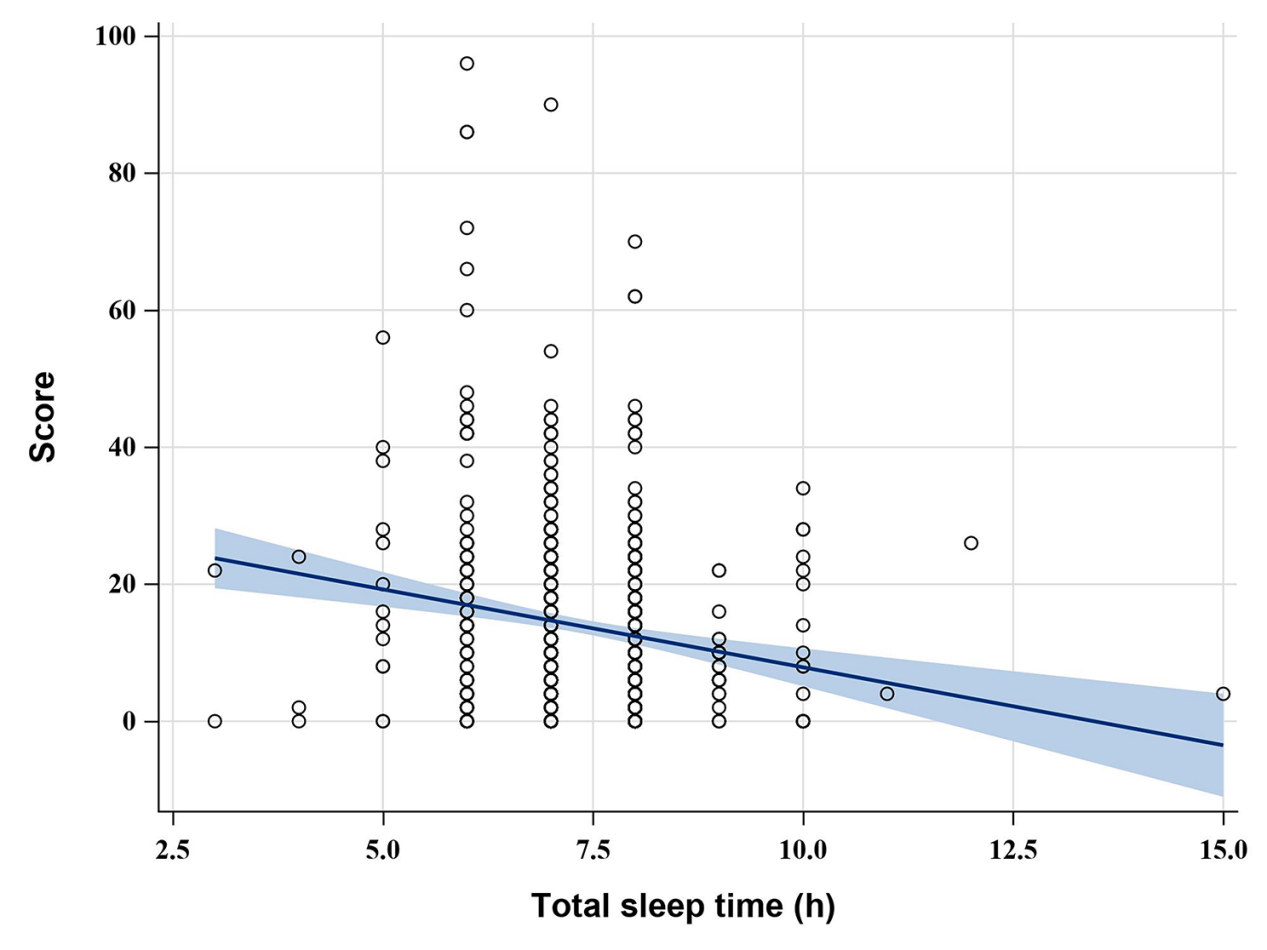
Total eye discomfort score and daily sleep duration

In this study, the degree of eye discomfort (“no eye discomfort”, “mild eye discomfort”, “moderate eye discomfort”, “severe eye discomfort “) as the dependent variable, and the cumulative value of the lower ordered value as the modeled probability; the variables that were statistically significant and professionally considered significant in the univariate analysis were used as independent variables to fit the ordered logistic regression model. The model parallelism test P = 0.126 indicated that the model was applicable to ordered logistic regression. The results of model fitting showed that gender, region, frequent use of eye drops, sleep time, total time of VDT use per day were the risk factors affecting the degree of eye discomfort, and the effects of the remaining factors were not statistically significant (1). Gender: OR _gender: male_ = 1.48, P = 0.011, indicating that males were 1.48 times more likely to have mild eye discomfort than females; (2) Geography: OR _geography: central region_ = 1.68, P = 0.011; OR _region_: eastern region = 1.52, P = 0.033, indicating that the likelihood of milder eye discomfort in the central region is 1.68 times higher than in the western region, and the likelihood of mild eye discomfort in the eastern region is 1.52 times higher than in the western region, with statistically significant differences between different altitudes, as shown in Fig. 1; (3) Frequent use of eye drops such as artificial tears: OR _frequent use of eye drops: yes_ = 0.26, P < 0.001, indicating that infrequent use of eye drops was 1.26 times more likely to cause mild eye discomfort than frequent use of eye drops; (4) Daily sleep duration: OR _sleep time_ = 1.25, P = 0.033, indicating that for every 1 h increase in daily sleep time, the possibility of mild eye discomfort increased by 25%; (5) Total time of VDT use per day: OR _total time of VDT use per day_ = 0.91, P < 0.001, indicating that for every 1 h increase in total daily VDT duration, the likelihood of mild eye discomfort increased by 9%, see Table 3 for details. At the same time, with the increase of daily VDT use, the total score of ocular discomfort symptoms increased significantly and the degree of eye discomfort gradually worsened, and there was a positive correlation between the two; while with the increase of daily sleep time, the total score of ocular discomfort symptoms decreased significantly, and the degree of eye discomfort decreased significantly, and there was a negative correlation between the two, as shown in Figs. 2 and 3.

**Table 3:** Multifactorial analysis of different degrees of eye discomfort

Variable	估计	SE	Wald	*P*	*OR*(95%*CI*)
**Intercept**(No eye discomfort) Intercept(Mild eye discomfort) Intercept(Moderate eye discomfort) **Gender**	-2.23 -1.14 2.65	0.60 0.59 0.64	13.77 0.00 17.36	< 0.001 0.947 < 0.001	
Female					1.00(ref)
Male	0.39	0.15	6.42	0.011	1.48(1.09 ~ 2.00)
**Region**					
West region					1.00(ref)
Middle region	0.52	0.21	6.40	0.011	1.68(1.12 ~ 2.52)
East region	0.42	0.20	4.52	0.033	1.52(1.03 ~ 2.25)
**Wearing contact lenses for more than 2 h per day**					
No					1.00(ref)
Yes	-0.16	0.28	0.31	0.580	0.86(0.49 ~ 1.48)
**Frequent use of eye drops**					
No					1.00(ref)
Yes	-1.35	0.30	19.90	< 0.001	0.26(0.14 ~ 0.47)
**Sleep time(h)**	0.22	0.07	9.14	0.003	1.25(1.08 ~ 1.44)
**Total time of VDT use per day(h)**	-0.09	0.02	17.13	< 0.001	0.91(0.87 ~ 0.95)
**Total time per VDT use(h)**	-0.02	0.03	0.32	0.573	0.98(0.93 ~ 1.04)

## Discussion

### Risk factors associated with the degree of eye discomfort

#### Altitude

In this survey, it was found that the degree of eye discomfort varied from region to region, and the possibility of developing severe eye discomfort gradually increased as the altitude gradually increased from east to west. College students living in the western region are significantly more likely to develop severe eye discomfort than those living in the central and eastern regions.

With the gradual increase in altitude, a number of physiological and pathological changes may occur that are closely related to the unfavorable environmental conditions associated with high altitude [36]. These unfavorable environmental conditions include low air pressure, low oxygen partial pressure, low humidity, low precipitation, strong wind, high UV radiation, etc. The various unfavorable factors brought about by relatively high altitude may lead to accelerated tear evaporation, which may result in an increase in the prevalence and degree of eye discomfort [37].

The high prevalence and severity of eye discomfort at high altitude is a health issue of great concern to ophthalmologists. The inevitable increase in the use of video terminals during the COVID-19 period, due to the long time closed at home or school, has led to the emergence and even the aggravation of eye discomfort. And to a large extent, allowing this eye discomfort to occur and progress, it will most likely lead to the emergence of organic dry eye disease.

To address this phenomenon, it is important to provide reasonable and effective health guidance and proper interventions for outpatients in ophthalmology, including college student populations. This includes many aspects such as the use of corneal contact lenses, pre and post-operative considerations and risks of refractive surgery, and the choice of artificial tears or dry eye treatment modalities. Medical staff should be trained to be sensitive and alert to dry eye to facilitate early recognition of symptoms and timely and accurate treatment, and early detection and timely management can effectively prevent vision-threatening complications resulting from severe dry eye disease.

#### Sleep duration

This investigation found that the shortening of sleep time had a significant effect on the degree of eye discomfort: as sleep time shortened, the likelihood of severe eye discomfort increased significantly, with a significant positive correlation between the two.

Sleep status is an important measure of well-being and health, and sleep disorders can cause abnormal brain function and increase the incidence of related diseases such as hypertension, diabetes, and dry eyes [38–40]. Eye diseases are associated with sleep disorders in many ways, including a variety of conditions such as poor sleep quality as well as short sleep duration [41]. However, both poor sleep quality and short sleep time can seriously affect our physical and mental health and reduce our quality of life [42]. The COVID-19 pandemic has stolen our social space and also affected our sleep to varying degrees, which has had a significant impact on global mental health [21]. It has been suggested that sleep deprivation may impair lacrimal gland function and induce eye discomfort, leading to increased levels of eye discomfort that may be alleviated with adequate rest, while the presence of eye discomfort may also lead to decreased sleep quality, creating a vicious cycle between the two [43, 44].

2016–2017 Xiaoning Yu et al. in a large community-based study similarly found a strong correlation between poor sleep quality and increased severity of dry eye, and that improving sleep quality and prolonging effective sleep time slowed the progression of dry eye, while at the same time, if eye discomfort symptoms were effectively relieved, then sleep disturbances could be adequately improved [38, 45]. In response to this phenomenon, when ophthalmologists are faced with patients with eye discomfort of different etiologies, especially some of those who have had poor results with conventional treatments, including artificial tears, lid gland massage, and other eye discomfort treatment measures, they recommend that patients undergo neurology and sleep consultation and treatment to address their underlying problems in order to possibly improve the cure rate of eye discomfort and dry eye to a greater extent.

#### Duration of VDT use

The present investigation found that an increase in the length of VDT use led to an increase in the severity of eye discomfort, with a positive correlation between the two. In a study of 4,393 young and middle-aged Japanese office workers, Miki Uchino et al. found that VDT use for more than 4 h was significantly associated with an increased risk of DED, which is consistent with the findings of this paper [46]. The reason for this analysis may be that reduced blink frequency and incomplete blinking when using close objects such as VDT reduces tear secretion and discharge, accelerated evaporation of tear film and tear film instability, leading to the appearance and extent of eye discomfort [47–49]. Therefore, minimizing the working time with near objects such as VDT may be beneficial to the eye health of college students. However, further studies may be needed to clarify the exact optimal time and specific distance for close work.

#### Gender

While much of the literature mentions the influence of gender on the prevalence of eye discomfort, this study found that younger college students also had a greater degree of eye discomfort among women. As with many of these findings, women have been repeatedly mentioned as an important risk factor for the progression of eye discomfort [50, 51], and analysis of this may be attributed to the influence of sex hormones. It has been shown that the meibomian gland is an androgen target organ and androgen deficiency may promote the development of meibomian gland dysfunction as well as eye discomfort [52].

At the same time, we consider that the frequency of cosmetics use in young women, especially eye cosmetics, increases significantly compared to men, and that the use of eye cosmetics may lead to problems such as blockage of the lid gland opening due to inadequate makeup removal, inadequate cleaning, product quality problems, and other factors, which may also lead to a significant increase in the prevalence and extent of dry eye [53], but its fact depends on further expansion of the sample size according to the actual situation investigation and analysis.

#### Frequent use of eye drops

The statistical findings of this study showed that patients who frequently used eye drops such as artificial tears were more likely to develop severe eye discomfort. We believe the reasons for this are that there is a wide variety of topical drug preparations on the market regarding artificial tears and other topical drugs, and that there is a lack of standardized purchasing channels and varying quality. The potential damage to ocular tissues is becoming apparent: most of the commercially available eye drops contain different types and concentrations of preservatives and excipients, which have synergistic damaging effects. The abuse of ocular preparations by patients without regular physician’s orders may pharmacogenetically induce epithelial damage and alterations in the ocular surface microbiome, leading to an increase in the degree of eye discomfort [54]. Therefore, it is important to have a clear understanding of drug toxicity when providing medication instruction to eye discomfort patients to avoid drug-derived ocular tissue damage.

## Conclusion

High altitude, women, shorter sleep time and longer total daily VDT use are risk factors associated with the degree of eye discomfort, where the likelihood of developing severe eye discomfort gradually decreases with increasing sleep duration and gradually increases with increasing total VDT use.

Prior to the COVID-19 pandemic, the socioeconomic burden caused by eye discomfort was already trending high [55, 56], and the social status quo brought about by the COVID-19 has undoubtedly exacerbated the impact on the severity and prevalence of eye discomfort especially dry eye. The symptoms associated with developing dry eye disease such as eye fatigue, eye pain, and even headache and sleep disorders may further lead to reduced work efficiency, reduced quality of life, and even increased mental health burden, thus increasing the socioeconomic burden. As a high-risk group for dry eye disease during the epidemic, it is especially important to understand the risk factors associated with the disease in order to provide targeted and effective treatment.

Through this investigation and analysis of the degree of eye discomfort syndrome caused by the use of VDT among college students in different altitudes, it can help us to formulate personalized prevention and treatment plans for patients attending clinics in different altitudes, so as to avoid and reduce the damage to physical and mental health and social and economic losses caused by the improper use of video terminals.

## Data Availability

All data generated or analyzed during this study are included in this published article.

## References

[1] Blehm C, Vishnu S, Khattak A, Mitra S, Yee RW. Computer vision syndrome: a review. Surv Ophthalmol. 2005 Jun;50(3):253–62. 10.1016/j.survophthal.2005.02.008 15850814

[2] Randolph SA. Computer Vision Syndrome. Workplace Health Saf. 2017 Jul;65(7):328. 10.1177/2165079917712727 28628753

[3] Computer vision syndrome. (Digital eye strain) [Internet]. [cited 2022 Oct 10]. Available from: https://www.aoa.org/healthy-eyes/eye-and-vision-conditions/computer-vision-syndrome?sso=y

[4] Gowrisankaran S, Sheedy JE (2015). Computer vision syndrome: a review. Work.

[5] Thomson WD. Eye problems and visual display terminals–the facts and the fallacies. Ophthalmic Physiol Opt. 1998 Mar;18(2):111–9. 9692030

[6] Smith MJ, Cohen BG, Stammerjohn LW. An investigation of health complaints and job stress in video display operations. Hum Factors. 1981 Aug;23(4):387–400. 10.1177/001872088102300402 7275107

[7] Sheedy JE. Vision problems at video display terminals: a survey of optometrists. J Am Optom Assoc. 1992 Oct;63(10):687–92. 1430742

[8] Dain SJ, McCarthy AK, Chan-Ling T. Symptoms in VDU operators. Am J Optom Physiol Opt. 1988 Mar;65(3):162–7. 10.1097/00006324-198803000-00004 3364524

[9] Zhang S, Hong J. Risk factors for Dry Eye in Mainland China: a Multi-Center Cross-Sectional Hospital-Based study. Ophthalmic Epidemiol. 2019 Dec;26(6):393–9. 10.1080/09286586.2019.1632905 31218906

[10] Song P, Xia W, Wang M, Chang X, Wang J, Jin S, et al. Variations of dry eye disease prevalence by age, sex and geographic characteristics in China: a systematic review and meta-analysis. J Glob Health. 2018 Dec;8(2):020503. 10.7189/jogh.08.020503 PMC6122008 30206477

[11] Xiaoyun M, Lihong Y, Shuna G. Epidemiological characteristics of dry eye of video terminal work population. Chin J Optometry Ophthalmol Visual Sci. 2014 Sep;25(09):527–31.

[12] Zhenyu W, Hanruo Liu Q, Liang (2020). Advances on the epidemiology of the dry eye. Chin J Ophthalmol (Electronic Edition).

[13] The Definition and Classification of Dry Eye Disease: Report of the Definition and Classification Subcommittee of the International Dry Eye Workshop (2007). The Ocular Surface. 2007 Apr 1;5(2):75–92. 10.1016/s1542-0124(12)70081-2 17508116

[14] Liu NN, Liu L, Li J, Sun YZ (2014). Prevalence of and risk factors for dry eye symptom in mainland china: a systematic review and meta-analysis. J Ophthalmol.

[15] Jin Y, Yuqing D, Peng X. Striving to promote precise diagnosis of dry eye. Chin J Ophthalmol 2022 Feb 11;58(02):85–9. 10.3760/cma.j.cn112142-20211122-00558 35144347

[16] Expert Consensus on Clinical Management of Dry Eye (2013). Chin J Ophthalmol 2013 Jan.

[17] Lingyi L, Jing L, Zuguo L (2019). Focusing on the functional dry eye. Chin J Ophthalmol.

[18] Zuguo L, Hua W (2018). Focusing on the management of chronic dry eye disease. Chin J Ophthalmol.

[19] Le Q, Ge L, Li M, Wu L, Xu J, Hong J, et al. Comparison on the vision-related quality of life between outpatients and general population with dry eye syndrome. Acta Ophthalmol. 2014 Mar;92(2):e124–132. 10.1111/aos.12204 23901943

[20] van der Vaart R, Weaver MA, Lefebvre C, Davis RM. The association between dry eye disease and depression and anxiety in a large population-based study. Am J Ophthalmol. 2015 Mar;159(3):470–4. 10.1016/j.ajo.2014.11.028 PMC4329250 25461298

[21] COVID-19 Mental Disorders Collaborators. Global prevalence and burden of depressive and anxiety disorders in 204 countries and territories in 2020 due to the COVID-19 pandemic. Lancet. 2021 Nov;6(10312):1700–12. 10.1016/S0140-6736(21)02143-7 PMC8500697 34634250

[22] Clayton JA, Dry Eye N, Engl JM. 2018 Jun 7;378(23):2212–23. 10.1056/NEJMra1407936 29874529

[23] Liu B, Jiang S, Li Z, Wang Y, Zhou D, Chen Z (2022). Investigation and analysis of Eye Discomfort caused by Video Display Terminal Use among Medical Students studying at high-altitude regions. Front Public Health.

[24] Fang Rukang. Topography of China [Internet]. Beijing: Commercial Press. ; 1995 [cited 2022 Nov 10]. Available from: https://xueshu.baidu.com/usercenter/paper/show?paperid=df30301ef3eb055ef6a2d0e7d6531331&site=xueshu_se.

[25] Karaküçük S, Mirza GE. Ophthalmological effects of high altitude. Ophthalmic Res 2000 Feb;32(1):30–40. 10.1159/000055584 10657753

[26] Fjaervoll H, Fjaervoll K, Magno M, Moschowits E, Vehof J, Dartt DA et al. The association between visual display terminal use and dry eye: a review. Acta Ophthalmol. 2021 Oct 25. 10.1111/aos.15049 34697901

[27] Marelli S, Castelnuovo A, Somma A, Castronovo V, Mombelli S, Bottoni D, et al. Impact of COVID-19 lockdown on sleep quality in university students and administration staff. J Neurol. 2021 Jan;268(1):8–15. 10.1007/s00415-020-10056-6 PMC7353829 32654065

[28] Koh S, Rhee MK. COVID-19 and Dry Eye. Eye Contact Lens 2021 Jun 1;47(6):317–22. 10.1097/ICL.0000000000000797 33990103

[29] National Education Development Statistics Bulletin. 2020 - Ministry of Education of the People’s Republic of China Government Portal [Internet]. [cited 2022 Jun 14]. Available from: http://www.moe.gov.cn/jyb_sjzl/sjzl_fztjgb/202108/t20210827_555004.html

[30] Li S, He J, Chen Q, Zhu J, Zou H, Xu X. Ocular surface health in Shanghai University students: a cross-sectional study. BMC Ophthalmol. 2018 Sep 12;18(1):245. 10.1186/s12886-018-0825-z PMC6134707 30208892

[31] Okumura Y, Inomata T, Iwata N, Sung J, Fujimoto K, Fujio K et al. A Review of Dry Eye Questionnaires: Measuring Patient-Reported Outcomes and Health-Related Quality of Life. Diagnostics (Basel). 2020 Aug 5;10(8):E559. 10.3390/diagnostics10080559 PMC7459853 32764273

[32] Wolffsohn JS, Arita R, Chalmers R, Djalilian A, Dogru M, Dumbleton K, et al. TFOS DEWS II Diagnostic Methodology report. Ocul Surf. 2017 Jul;15(3):539–74. 10.1016/j.jtos.2017.05.001 28736342

[33] Amparo F, Schaumberg DA, Dana R. Comparison of two questionnaires for Dry Eye Symptom Assessment: the ocular surface Disease Index and the Symptom Assessment in Dry Eye. Ophthalmology. 2015 Jul;122(7):1498–503. 10.1016/j.ophtha.2015.02.037 PMC4485570 25863420

[34] Schiffman RM, Christianson MD, Jacobsen G, Hirsch JD, Reis BL. Reliability and validity of the ocular surface Disease Index. Arch Ophthalmol. 2000 May;118(5):615–21. 10.1001/archopht.118.5.615 10815152

[35] Ozcura F, Aydin S, Helvaci MR. Ocular surface disease index for the diagnosis of dry eye syndrome. Ocul Immunol Inflamm. 2007 Oct;15(5):389–93. 10.1080/09273940701486803 17972223

[36] Basnyat B, Cumbo TA, Edelman R (2000). Acute medical problems in the Himalayas outside the setting of altitude sickness. High Alt Med Biol.

[37] Th M. G T. Going to high altitude with preexisting ocular conditions. High altitude medicine & biology [Internet]. 2003 Winter [cited 2022 Jul 26];4(4). Available from: https://pubmed.ncbi.nlm.nih.gov/14672545/10.1089/152702903322616173 14672545

[38] Ayaki M, Tsubota K, Kawashima M, Kishimoto T, Mimura M, Negishi K. Sleep Disorders are a Prevalent and Serious Comorbidity in Dry Eye. Invest Ophthalmol Vis Sci. 2018 Nov 1;59(14):DES143–50. 10.1167/iovs.17-23467 30481819

[39] St-Onge MP, Grandner MA, Brown D, Conroy MB, Jean-Louis G, Coons M et al. Sleep Duration and Quality: Impact on Lifestyle Behaviors and Cardiometabolic Health: A Scientific Statement From the American Heart Association. Circulation. 2016 Nov 1;134(18):e367–86. 10.1161/CIR.0000000000000444 PMC5567876 27647451

[40] Cappuccio FP, D’Elia L, Strazzullo P, Miller MA. Quantity and quality of sleep and incidence of type 2 diabetes: a systematic review and meta-analysis. Diabetes Care. 2010 Feb;33(2):414–20. 10.2337/dc09-1124 PMC2809295 19910503

[41] Lee SSY, Nilagiri VK, Mackey DA. Sleep and eye disease: a review. Clin Exp Ophthalmol. 2022 Apr;50(3):334–44. 10.1111/ceo.14071 PMC9544516 35263016

[42] Chen X, Gelaye B, Williams MA. Sleep characteristics and health-related quality of life among a national sample of american young adults: assessment of possible health disparities. Qual Life Res. 2014 Mar;23(2):613–25. 10.1007/s11136-013-0475-9 PMC4015621 23860850

[43] Li S, Ning K, Zhou J, Guo Y, Zhang H, Zhu Y et al. Sleep deprivation disrupts the lacrimal system and induces dry eye disease. Exp Mol Med. 2018 Mar 2;50(3):e451. 10.1038/emm.2017.285 PMC5898890 29497171

[44] Wu M, Liu X, Han J, Shao T, Wang Y. Association Between Sleep Quality, Mood Status, and Ocular Surface Characteristics in Patients With Dry Eye Disease. Cornea. 2019 Mar;38(3):311–7. 10.1097/ICO.0000000000001854 30614900

[45] Yu X, Guo H, Liu X, Wang G, Min Y, Chen SHS et al. Dry eye and sleep quality: a large community-based study in Hangzhou. Sleep. 2019 Oct 21;42(11):zsz160. 10.1093/sleep/zsz160 31310315

[46] Uchino M, Schaumberg DA, Dogru M, Uchino Y, Fukagawa K, Shimmura S et al. Prevalence of dry eye disease among japanese visual display terminal users. Ophthalmol 2008 Nov;115(11):1982–8. 10.1016/j.ophtha.2008.06.022 18708259

[47] Tsubota K, Toda I, Nakamori K. Poor illumination, VDTs, and desiccated eyes. Lancet. 1996 Mar;16(9003):768–9. 10.1016/s0140-6736(96)90122-1 8602034

[48] Argilés M, Cardona G, Pérez-Cabré E, Rodríguez M. Blink rate and incomplete blinks in six different controlled Hard-Copy and Electronic Reading Conditions. Invest Ophthalmol Vis Sci. 2015 Oct;56(11):6679–85. 10.1167/iovs.15-16967 26517404

[49] Tsubota K, Nakamori K. Dry eyes and video display terminals. N Engl J Med 1993 Feb 25;328(8):584. 10.1056/NEJM199302253280817 8426634

[50] Matossian C, McDonald M, Donaldson KE, Nichols KK, MacIver S, Gupta PK. Dry Eye Disease: consideration for women’s Health. J Womens Health (Larchmt). 2019 Apr;28(4):502–14. 10.1089/jwh.2018.7041 PMC6482917 30694724

[51] García-Ayuso D, Di Pierdomenico J, Moya-Rodríguez E, Valiente-Soriano FJ, Galindo-Romero C, Sobrado-Calvo P. Assessment of dry eye symptoms among university students during the COVID-19 pandemic. Clin Exp Optom. 2021 Jul;19:1–7. 10.1080/08164622.2021.1945411 34279190

[52] Sullivan DA, Sullivan BD, Evans JE, Schirra F, Yamagami H, Liu M, et al. Androgen deficiency, meibomian gland dysfunction, and evaporative dry eye. Ann N Y Acad Sci. 2002 Jun;966:211–22. 10.1111/j.1749-6632.2002.tb04217.x 12114274

[53] Ng A, Evans K, North R, Purslow C. Eye cosmetic usage and associated ocular comfort. Ophthalmic Physiol Opt. 2012 Nov;32(6):501–7. 10.1111/j.1475-1313.2012.00944.x 23057565

[54] Zhang X, Qu MVJ, He Y, Ou X, Bu S. J, Dry Eye Management: targeting the Ocular Surface Microenvironment. Int J Mol Sci 2017 Jun 29;18(7):E1398. 10.3390/ijms18071398 PMC5535891 28661456

[55] The epidemiology of dry eye disease: report of the Epidemiology Subcommittee of the International Dry Eye WorkShop. (2007). Ocul Surf. 2007 Apr;5(2):93–107. 10.1016/s1542-0124(12)70082-4 17508117

[56] Nj F. Impact of dry eye disease and treatment on quality of life. Current opinion in ophthalmology [Internet]. 2010 Jul [cited 2022 Aug 4];21(4). Available from: https://pubmed.ncbi.nlm.nih.gov/20467319/10.1097/ICU.0b013e32833a8c15 20467319

